# Outline of the Principal Diseases of Females

**Published:** 1838-10-01

**Authors:** 


### DISEASES OF FEMALES.

Outlines of the principal Diseases of Females. Chiefly for the Use of Students. By *Fleetwood Churchill*, M.D. &c. &c. &c. Dublin, 1838. Pp. 402.

There is nothing, in the whole history of medicine, more surprising, or more gratifying, than the improvements which have been made in the study of that science, in the metropolis of this country alone, during the last ten years.

Formerly there were no dissuasives from idleness, no encouragements to diligence, no motives to industry, beyond those supplied by the prospect of a couple of examinations, and a fear of rejection by the respective Boards of Examiners. Formerly there was hardly such a thing known or heard of as periodical examinations by the several teachers of their several classes. Here one, and there another—to the infinite annoyance of the majority of their pupils, practised a better method, and made a point of guaging the progressive attainments of the young men attending their lectures, by the institution of occasional examinations. They formed exceptions to the general rule, which consisted in giving lectures, and no more. If the pupils were attentive to those lectures, well; if they were constant attendants, as well as attentive listeners, better still; and if they took notes at the time, and sat down afterwards to the study of those notes, best of all. They acquired, there can be no doubt, much valuable information. And are now, in the practice of their profession reaping the benefit of their teachers' instructions, and of their own industry. But, until the dreaded hour of trial came, there was no one who either knew or cared whether the student was really profiting by the lectures he attended; or whether those lectures were not entirely thrown away upon him.

The case is widely different now; and instead of class examinations being exceptions, they are the present rule, so far as we are acquainted, in every school in London, and in almost every department of medical education. The advantage accruing from this has been two-fold—by it the attention of the students, for the time being, has been fixed to the subject matters of the daily lectures—their energies roused to make the matters taught them their own.

But not only has the mode of teaching been subjected to improvements,—and the above is not the only improvement which has been introduced, like an after-thought, into the course of medical instruction;—the science of medicine has been made to enlarge her academical boundaries—and branches of knowledge have been included in the more comprehensive curriculum of medical studies, which were previously studied apart, as collateral, but by no means necessary; or were not studied at all.

Two of these branches to which we more particularly refer, are, Obstetricy—and Forensic Medicine. Every candidate for the chirurgical diploma must now furnish proofs of having studied them. Every one seeking the Apothecaries' Licence, may expect to have his knowledge of them tested, by competent examiners.

We hail the improvements that have been made in the mode of study, as blessings which have come upon our country, and our own especial class of society. We rejoice in the wider extension of professional requirements, because, assenting to the axiom of Lord Bacon, that "knowledge is power," and further believing, that not money, but professional knowledge, is the professional man's best capital, we feel persuaded that the day is not far distant when the honours which wait on power, and the respect which is paid to wealth, will descend in refreshing showers upon the medical profession, at present a profession—less honoured, less respected, and worse requited than either of its learned sisters. A profession, where studies the most laborious, only lead to labours the most unprofitable. A profession, the members of which work harder, we speak it advisedly, than any men on the face of the whole earth—and, in England at least, which boasts herself of being in advance of all other countries, are worse paid, in reference to their work, than any. Witness only one instance. The onerous duties which devolve upon parochial medical officers—and the compensation doled out to them with niggard hand, by the respective Boards of Guardians at the instance of three Poor Law Commissioners, who know nothing of the study or practice of medicine, and can know nothing unless a fit of the gout or colic should some day make them rest from their labours, and move the bowels of their compassion to feel—if not for others—for themselves.

> To each his sufferings:—all are men
> Condemned alike to groan;
> The feeling—for another's pain:
> The unfeeling—for his own.

But this is no time for casting more than a hurried glance at the past history of our profession. It needs further improvements, and it's motto should be "Forward!"

We have been led to make these observations in considering the subject of female diseases—and we give them with less hesitation to our readers, seeing that, important as is the study of the complaints to which females are subject—and scarcely less difficult than important—there is, at the present moment, a crying defect in the mode of prosecuting it, and a corresponding deficience of the means at the command of the various teachers, for communicating clinical instruction, in a class of diseases, certainly as numerous as any—and as certainly of equal moment with any—whether we regard the well-being of the patient—or the success of the practitioner.

What is wanted in every school of medicine in the kingdom is an additional ward or two for cases of purely female complaints—where they may be seen by students.*

---

* Some of the London hospitals have such additions. The Middlesex and Guy's, for example. In the former, there is a ward for cancer patients, another for lying-in-women, and a physician-accoucheur attached to the hospital. In the latter, there is the Petersham Ward—the limited sphere of usefulness of which may be gathered from Dr. Ashwell's statements in the 6th number of the Guy's Hospital Reports. From these it appears, that only 82 cases were received into it, from December 1836 to October 1837. The extent to which that usefulness might be enlarged is evident from the fact that the obstetric out-patients of Guy's Hospital, from October 1836 to October 1837, amounted to 497.

Such an arrangement must, sooner or later, be made. The present state of medical science requires it, the future wants of medical students will render it imperative. Where all are rightly emulous to be excellent, which shall take the lead in this matter, it is not for us to predict—that the lead will soon be taken by some one or other of our many medical schools, we are confident. That it cannot be taken too soon, we know—and hope to persuade our readers likewise, by bringing before them in a series of analytical articles, "the Principal Diseases of Females," shewing their number, their prevalence and their importance.

The valuable work of Dr. Churchill enables us to introduce the subject now, and we hope to continue it more in detail hereafter. The work of Dr. Churchill, however, is not yet complete, its author waiting the approval of the present volume by the public voice of the profession, before publishing another on the Diseases of Women during Gestation, and in Childbed. That the publication of that volume may not be unnecessarily delayed, we hasten to register our vote in his favour; and to give our own readers the benefit of as full an analysis as our limits will admit.

Dr. Churchill divides his subject into two parts. The first, treating of "Diseases of the External Genitals." The second, of—"Diseases of the Internal Genitals." Which last is further subdivided into—1st. "Diseases of the Vagina. 2d. "Diseases of the Uterus." 3d. "Diseases of the Fallopian Tubes." 4th. "Diseases of the Ovaries." A very convenient division for the study of those diseases *in books*—but far from the most useful mode of classifying them, for observation at the bed-side.

We like Dr. Good's better, (Study of Medicine, Vol. IV. ed. first.) He divides his class Genetica into three orders, *Cenotica*, or diseases affecting the fluids—*Orgastica*, those affecting the orgasm—and *Carpotica*, or those affecting the impregnation. But this division is likewise faulty, and especially so with reference to the principles adopted by its very learned author, in his Nosology generally. And, while it banishes some diseases to other classes, it admits others, which belong to another place.

Dr. Mackintosh, in the eighth part of his work, treats of these diseases under another arrangement, if indeed that can be called arrangement which has scarcely a semblance of it. For example, his seventh chapter, like the first part of Dr. Churchill's volume, is entitled "Diseases of the Labia, and External Parts in the Female." The title of the *ninth* chapter is, "Diseases of the Uterus, connected with Inflammatory Action"—of the *tenth*, "Prolapsus of the Uterus—Retroversion of the Uterus—*Polypus of the Vagina and Uterus*"—of the *eleventh*, "Tubercles of the Uterus—Bony Concretions—Hydatids—Aqueous and Flatulent Discharges"—of the *twelfth*, "Fluor Albus and Leucorrhœa"—of the *thirteenth*, "Diseases of Menstruation"—and of the *fourteenth*, "Diseases of the Ovaria."

In Dr. Marshall Hall's skeleton work—"Principles of the Theory and Practice of Medicine," there is an attempt at classification of the diseases, not only incidental, but peculiar to females, which simplifies their division very materially, and renders it more natural than either of the others. Dr. Hall arranges them under three classes, Diseases of the Uterus—Ovaria—and Mammæ. And distributes the first class into two orders—the organic and the functional. The following admirable remarks, with a characteristic anecdote of the great GREGORY, introduces the subject in Dr. Hall's volume, and we cannot do better than cite them in their author's own words:—

"As the kidney, the bladder, the prostate, form a series or system of organs, the diseases of which mutually induce or aggravate each other; so do, in an especial manner, the uterus, the ovaria, the mammæ, &c. It is still an important inquiry how far remedies applied to one part of the series may relieve disordered actions in another. And the bond of connexion which binds these several organs amongst each other, and with the whole system, still affords a subject of deep interest for renewed inquiry. There is no question that the head is frequently affected by the condition of the uterine system. This is seen in nymphomania. On the other hand, phthisis disposes to conception, and this frequently checks the progress of phthisis. And cancer occurs simultaneously in the mamma and in the uterus. These connexions are still more readily traced in the physiology of the uterine system.

Dr. Gregory was consulted, in the town of Ayr, in the case of a lady who had repeatedly miscarried, with dreadful hæmorrhage, in spite of every remedial means which could be devised by the first medical authorities in Scotland. Dr. Gregory saw the patient on one of these occasions; he prescribed for the hæmorrhagy, and, when this had been arrested, and the patient had sufficiently recovered, he examined the state of the mammæ, found them distended with milk, and directed a lusty infant to be applied, and nursed for nine months. The course of the uterine blood was directed into another channel, the lady became pregnant, the mother of a living child, and ultimately of a numerous family, her labours being unattended by hæmorrhage!

"This history," Dr. Hall goes on to remark, "bears the stamp of genius. The fact itself is full of interest, and perhaps of more extensive application than may appear at first sight. May not the disposition to uterine hæmorrhagy, in other instances, be prevented by attention to the due adjustment of the mode and period of lactation?

I have throughout these sketches called the attention to one important principle—that the diseases are not simple—not the affections of single organs—but of *systems*; and I again take the liberty of repeating this remark in connexion with the diseases of the uterine organs."—*Hall*, 426-7-8.

Neither adopting one or other of the above attempts at classification, we propose to consider the diseases in question under two heads—1st. ORGANIC DISEASES OF THE UTERUS AND UTERINE SYSTEM; and 2nd, FUNCTIONAL DISORDERS OF THE UTERUS AND UTERINE SYSTEM; which will embrace every particular complaint, both before, during, and subsequent to gestation.

The first organic disease of the uterine system in the unimpregnated state, to which our attention is called, is, inflammation. The varieties of inflammation to which it is subject are occasioned by the parts in which that disease has its seat. Whether they be the external labia—the vulva—the vagina—the uterus and its appendages—or the peritoneal covering of the uterus.

### I. PHLEGMONOUS INFLAMMATION OF THE EXTERNAL LABIA PUDENDI.

This result of blows, falls, forcible intercourse, and other injuries of any kind, occurs frequently before, occasionally during, and frequently after pregnancy.

Its presence is known to the patient by increased heat and pain at its commencement, followed by swelling of the parts and a throbbing sensation, which generally render her incapable of motion, or compel her to remain at rest and quietness, by rendering every movement distressing. The practitioner discovers on examination, enlargement of one or both labia—a circumscribed hardness—intolerance of pressure—and a blush, more or less vivid, of inflammatory redness; and distinguishing it from tumefaction and from œdema of the labia, and from hernia, pronounces a favorable prognosis, and has only to adopt a simple treatment to ensure a successful result. *Rest*—leeches over the affected part—succeeded by hot fomentations and cataplasms—the use of the bidet night and morning—and mild aperients will sometimes dissipate the inflammation, where it has been attended to in time; but the looseness of texture hastens the suppurative process, and then the position must be altered from the recumbent to the upright—and the sooner the lancet lets out the secreted pus the better. There is no better application than a warm poultice thrice a-day afterwards—and all officious meddling with the wound occasioned by the lancet is to be deprecated. The practice of leaving the evacuation of the pus to nature as advocated by Denman, Burns, and Blundell, is happily becoming an antiquated and will soon be an exploded one. There is not one good reason to be assigned in its favour. There are many very sufficient objections against it.

*Case* 1.—M. C., æt. 18: was seduced by a policeman; a few days afterwards, the writer of this article saw, and examined her—there was extensive discolouration of the whole of the left labium—increased heat and swelling—the tumor fluctuated under the finger, and there was considerable constitutional disturbance, partly the result of agitation at the discovery of her weakness, partly the consequence of the injury. An incision was instantly made—fomentations and poultices were applied three times a day—a dose of compound jalap powder was administered—and the patient was well on the tenth day. In this case the inflammatory was changed from an unhealthy into a healthy process—the sides of the abscess adhered—the wound made by the lancet healed by the first intention—and the sufferer was well three weeks earlier than she would have been, had the suppuration been permitted to go on until the abscess opened spontaneously, when there would have been extensive ulceration, and proportionally delayed recovery. Dr. Mackintosh says rightly, that "mortification ought to be a very rare termination."

An inflammatory affection of the labia, from dirt and acrid discharges is sometimes met with among the poor. The cuticle is abraded, and a sero-purulent discharge oozes from the naked cutis, which becomes indurated—a circumscribed blush of inflammation surrounds it. The first thing to be enjoined in these cases is cleanliness, and that of all things is the most difficult to be enforced with some persons, whose skin, like the Ethiopians, never parts with its dinginess. Almost the only way to enforce it is to cover the part with a warm moist poultice—the change of this is attended with a removal of dirt—and the cause removed, the effect disappears. The following case (2) illustrates this affection. Mrs. D., æt. 33: the mother of several children and in a state of pregnancy, complained of pain and soreness in both labia—increased by walking—and accompanied with a discharge—the pain and the discharge together, driving her, as she said, mad. After a good deal of affected modesty she submitted to an examination—the filthiness of the parts was abominable—the stench intolerable. Seat yourself in hot water immediately—and keep in the bucket till I see you to-morrow, was the first prescription ; the following day the nastiness and stench were both removed. Fomentations and poultices ordered and constantly applied three times a day—on the twelfth day she was cured of the complaint, but not, it is feared, of the uncleanliness which caused it, and she will probably be again and again subject to its recurrence. The only internal remedies administered in her case, were saline purgatives and nauseating ptisans of tartar emetic.

II. Inflammation of the Mucous Membrane of the Vulva,

Occurring in infants as well as in adults—occasioned in the former by cold, mechanical injuries, the application of irritants, uncleanliness, and intestinal irritation: and caused in the latter by dirt. In children it is sometimes only a prevalent symptom of an epidemic disease. As it occurs in infants every practitioner ought to be acquainted with it. For fatal consequences have followed the mistake into which some have fallen, considering it a sequence of violent intercourse, instead of recognising it as a disease of no uncommon occurrence. Sir Astley Cooper was accustomed to call the particular attention of his surgical class to this disease, and most emphatically to caution them against falling into the mistake referred to. His benevolent wish that his remarks upon both might be echoed from one end of the kingdom to the other, we shall help him to accomplish.

"This is a disease," says Dr. Churchill, "occurring at all periods of life, but presenting considerable differences according to the age of the patient. In children it occupies the whole of the mucous membrane of the external genitals, sometimes, but rarely, spreading to the vagina, accompanied with a profuse puriform or milky discharge, with smarting, but not severe pain, and ending in resolution, ulceration, or gangrene. This is the *leucorrhœa infantilis* of authors. In adults, on the contrary, the inflammation is very often partial and circumscribed, with a slight colourless discharge, intense pain, and ending almost always in resolution, very rarely in ulceration, and, as far as my observations have gone, never in gangrene."

"The commencement of the disease is marked by local uneasiness, itching, and scalding on making water; the mucous membrane of the vulva is found inflamed and puffy, but, for some time, there is no discharge. The uneasiness felt by the child induces an attempt to relieve it by rubbing the part, which of course, aggravates the suffering, and increases the inflammation. At a more advanced stage, there is observed a colourless thin mucous discharge, speedily becoming more copious, thicker, and of a white or yellow colour. It is very often of an acrid character, and gives rise to a ring of inflammation and sometimes of excoriation of the skin at the margin of the vulva. If the labia be separated, the mucous membrane will be found more vascular and of a deeper colour than usual; but in very few cases does the inflammation extend up the vagina. The distress is increased with the progress of the disease—the smarting and scalding are very severe, and the little patient cannot walk without pain. It is very rare to find any constitutional disturbance, unless where this attack is but the local development of a general catarrh. Under ordinary circumstances the disorder is neither very tedious nor very obstinate, and, after running, a certain course, it terminates in resolution."—*Churchill*, p. 10.

Dr. Percival, Boivin, Dugès, Dr. Ferriar and Mr. Wood, of Manchester, and Dr. Mackintosh of Edinburgh, have all put cases on record of the above disease in its more malignant form. Dr. Percival's case, Medical Ethics, p. 103 and 231, is one deserving of mention, and ought not to be forgotten.

*Case* 3.—A little girl, four years old, was admitted an out-patient of the Manchester Infirmary, Feb. 11, 1791. The organs of generation were inflamed, sore, and painful. The child was reported to have been well until the day preceding, when she complained of pain on voiding her urine. She had slept two or three nights in the same bed with a boy, 14 years old, and had complained of his hurting her during the night. Leeches were applied, external applications and internal remedies administered in vain. The patient lingered a few weeks, becoming more and more debilitated, then died. A post-mortem inspection was instituted—an inquest held—the medical man's *opinion* received—upon that opinion, and the accompanying testimony of the mother—for *evidence* there could be none—a verdict of murder was returned against the boy with whom she had slept. And, but for the providential occurrence of similar cases in the immediate neighbourhood, about the same time, and the commendable candour of the surgeon in acknowledging his mistake upon the occurrence of those cases, the poor boy must have been executed for an offence, which it is probable he had never so much as contemplated.

The painfully interesting cases published by Mr. Wood, (Medico-Chirurgical Transactions, vol. vii. p. 84), have been long before the profession, and when first published, excited considerable attention. Dr. Ferriar "met with several instances of putrid fevers in young girls, accompanied with broad maculæ on the body and limbs, and a gangrenous state of the labia pudendi. The parts were greatly tumified and extremely painful. It was a very fatal complaint."—Medical Histories and Reflections, p. 169. And Dr. Mackintosh witnessed its occurrence as a sequel of measles in several cases. We give them in the author's own words with the view of their pathology taken by him.

*Case* 4.—"The first case of this affection that fell under my observation, occurred several years ago, in my dispensary practice. The patient, a girl of six years of age, when recovering from measles, during the progress of which there had been great gastro-intestinal irritation and diarrhœa, was seized with the disease of the pudendum, and died in the course of eight days. Every effort was made to obtain permission to examine the body, without success, but I saw sufficient to convince me that the child died, not so much from the effects of the external disease, as from inflammation, and perhaps ulceration, of the mucous membrane of the bowels.

*Case* 5.—Some time afterwards, I was asked by Dr. Moffit of the 7th Hussars, to see a child labouring under the disease at Peirshill Barracks; she was also attacked with it immediately after the recession of the eruption in measles, which had been mild, but attended with diarrhœa. The external inflammation, pain, and swelling of the pudendum, were fully as great as in the former case, and bore such a strong resemblance in its external character, that any one would have readily recognized the affection from a drawing in any portfolio. This child recovered under the use of poultices and fomentations, and the exhibition of gentle laxatives. Since then, several fatal cases have occurred in Edinburgh, and the appearances on dissection were such as to confirm the opinion I had previously entertained,—the mucous membrane of the bowels displaying extensive vascularity and ulceration, particularly in the ileum. In one of the cases, the ulcerations were numerous and extensive; and in the other, the mucous membrane was found thickened and spongy in many places, and in the usual progress towards ulceration, which would certainly have taken place had the child lived a few days longer."—*Mackintosh's Principles of Pathology, &c.*, vol. 2, p. 384.

The treatment of the milder form of this disease consists in fomentations—refrigerant and opiate injections—the prohibition of all stimulants—and the exhibition of mild aperients. In the more severe form, the treatment should commence with a purgative, and proceed with warm poultices. When ulceration begins, a decoction of bark, with the addition of aromatic confection, and of the tinctures of calumbo and opium, is recommended to be given internally, with wine.

In adults, inflammation of the vulva differs considerably from that already described as occurring in children. It is not so diffused—not so apt to run on to a breach of surface, and only gives rise to a discharge of transparent mucus. The pain is incalculably more severe. It usually terminates in resolution. Adhesion of the opposite surfaces may take place, but can only do so as a consequence of neglect. The treatment indicated, is more or less antiphlogistic. Rest, cleanliness, spare diet, empty bowels, emollient fomentations, and warm poultices, will generally effect a cure.

### III. INFLAMMATION OF THE MUCOUS MEMBRANE OF THE VAGINA, OR VAGINAL LEUCORRHŒA,

Commences with a sense of heat and soreness in the vagina; and an itchiness of the external parts; gradually increasing until pain and smarting are experienced, with a conjoint feeling of weight, tightness, and bearing down. Weight in the lower belly, and pain along the thighs, will also be felt, when the attack is violent. In a day or two after the above symptoms set in there is a discharge, varying in quantity, of a thin, colourless, sometimes acrid fluid, which shortly after becomes puriform, resembling cream in colour and consistence. On examination, made at the commencement of the disease, the mucous membrane of the vagina is found to be swollen and puffy—hot and tender—but without breach of surface. In most of the cases examined by Dr. Churchill, the vaginal portion of the cervix uteri was unaffected. The labia pudendi are occasionally swollen, the glands of the groin, more rarely, enlarged. As the disease advances, it parts with the acute inflammatory features, and assumes a chronic character, of which the principal sign is a profuse discharge. The heat and soreness are subdued—the swelling of the mucous membrane subsides.

The constitutional disturbance is proportioned to the severity of the attack; and, as well as the local symptoms, is allayed by the occurrence of the discharge.

It may be protracted from two days to a month; and treated promptly and judiciously, may terminate in resolution; or, neglected, slide into the chronic form.

Sir Charles M. Clarke deems it impossible to distinguish it from gonorrhœa. Yet the diagnosis may be essentially necessary to the domestic peace of a family. Ricord has supplied us, however, with a means of distinguishing the two diseases in some cases. Of 200 cases of gonorrhœa, examined by him with the speculum, two-thirds had an urethral discharge—an occurrence but seldom met with in leucorrhœa. He also found as the almost invariable attendant on gonorrhœa, that is, in 19 out of 20 cases, erosions or superficial ulcers of the mucous membrane covering the cervix uteri. The glands of the groin sympathize less with the discharge in leucorrhœa than with that of gonorrhœa. And not a little stress is to be laid upon the moral character and habits of the patient.

*Case* 6. Mrs. W——, six months married to her second husband. The mother of several children—and having suffered greatly from preternatural labours—after a widowhood of seven years, married. The week before her marriage she had menstruated—a fortnight after was troubled with a muco-purulent discharge, which her family medical man pronounced to be gonorrhœa. Suspicion attached to her husband—and the domestic peace of the parties was apparently destroyed. Month after month passed over, not only without improvement, in her condition, but with progressively increasing discharge—debility—and distress. And, after six months, the writer was called in. She had the anxious, chlorotic countenance; complained of great pain and swelling in the hypogastric region on the left side; restlessness through the nights, nausea and occasional sickness through the days; menstruation arrested from the date of her marriage; and her mind haunted with the idea of having been irrecoverably injured by her husband on their marriage. She denied being pregnant. The breasts were examined, and the areola were found distinctly indicative of a pregnant state. She was told so—but affirmed it could not be, and that she, of all women in the world, could not be mistaken. The vagina was inspected, and the judgment pronounced was, "you are not suffering under any disease communicated by your husband—but you are pregnant." In a week after her constitution rallied. In three weeks more, premature labour came on. She is at this time convalescent, with no discharge whatever from uterus or vagina. And, better still, is perfectly reconciled to her husband.

The treatment should be more or less antiphlogistic, in proportion to the intensity of the inflammation. Leeches to the vulva, followed by fomentations, and injections of hot solutions of the dilute acetate of lead, are principally to be depended on as external applications. Internally, nauseating doses of tartar emetic may be given with success. And, of course, rest, quiet, and a spare diet, should be enjoined. When there is much irritability, a solution of nitrate of silver may be applied with advantage.

When the use of the above remedies has been resorted to early, and persevered in diligently, without curing the disease, it is apt to assume the chronic form before alluded to.

In chronic vaginitis, the discharge has been ascribed to relaxation and debility:—that debility and relaxation may supervene upon a sharp attack of inflammation, is certain; and that a discharge resulting from inflammation in the first instance, may continue to flow when the inflammation is at an end, and even because it is at an end, we can also conceive; and that such is the case, moreover, in the chronic form of leucorrhœa: but we entirely agree with Dr. Churchill in tracing the disease in both its varieties to inflammation, attacking the mucous membrane of the vagina—and consider it of no small importance as affecting our practice. The chronic is caused by the same circumstances as the acute. The discharge however in the former differs from that in the latter: sometimes it is bland and colourless; sometimes brown and acrid. There is but little increased heat—little or no pain or tenderness. The inguinal glands are never affected. *If the discharge be profuse,* weakness is induced, and weariness felt, with aching pains in the back and loins;—*if it be long-continued,* dyspepsia follows.

Depletory measures are no longer, or but seldom necessary, when the acute has lapsed into the chronic vaginitis. Astringent lotions injected into the vagina. Opiates and tonics administered internally, will generally cure the disease. Leeches *may* be necessary at the beginning—when any inflammation is remaining they should be applied to the vulva, and the parts afterwards washed with fomentations.

It is sometimes complicated with uterine leucorrhœa—and Dr. Jewel has noticed a metastasis to the joints, in some cases, upon sudden suppression of the discharge.

### IV. INFLAMMATION OF THE GLANDULAR STRUCTURE OF THE MUCOUS MEMBRANE COVERING THE CERVIX UTERI.

This disease has been described by Sir C. M. Clarke, under the title of "the white discharge"—the peculiarity of which, and the state of the cervix and os uteri, are its distinguishing marks.

The symptoms are an aching sensation or pain in the back, and lower part of the abdomen—increased by motion, pressure, and sexual intercourse; a perfectly white-coloured, opake discharge; tenderness and puffiness of the cervix uteri. "In many instances, the white mucous discharge is much thicker than cream, having the tenacity of glue; and perhaps this is the state in which it came away from the cervix uteri. When the white opaque mucus possesses the tenacity just mentioned, it does not flow spontaneously, but it remains in the vagina, either until the exertions employed to empty the rectum squeeze out at the same time the contents of the vagina, or perhaps, by remaining in the vagina, it may, by mixing with the mucus of that part, become attenuated."—*Clarke on Diseases of Females,* vol. ii. p. 7.

"Judging from the local symptoms generally present, and from the resemblance which this white discharge has to the secretion from the glands in the mucous membrane of the neck of the womb under other circumstances, Sir C. Clarke concludes that it is this glandular apparatus which is the seat of the inflammation in this disease. There are seldom any constitutional symptoms present.

Sir C. M. Clarke throws out a hint as to the probability of this affection of the glandular apparatus being the precursor of more serious uterine disease, as carcinoma—a supposition which is strengthened by the greater frequency of the latter disease in glandular than in any other structure, and by the destruction of the cervix preceding that of any other part of the uterus in cancer." *Churchill, p.* 30.

The treatment, like that of the former diseases, is antiphlogistic. Dr. Churchill says he has found cupping over the loins by far the most efficacious mode of taking away blood. Might not a few leeches be introduced in a tube and applied to the cervix uteri, thereby attacking the disease directly?

The hip-bath twice a day—and injections of warm water thrown up the vagina three or four times a day, will furnish considerable relief. The bowels should be kept open—and, as there is generally irritability of the bladder present, the best form of purgative is castor oil, combined with a full dose of laudanum.

### V. Granular Inflammation of the Mucous Membrane of the Cervix Uteri.

The best account of this disease is given by Boivin and Dugès in their valuable work.

The granules are visible on the labia of the os, and on the external surface of the cervix uteri; and, when the result of acute inflammation, are few in number, about the size of peas, subpediculated, firm, and whitish; or, not larger than millet-seeds when they are numerous, without a pedicle—soft, and even vesicular; the parts are red and vascular, bleeding when touched; and there are pain and vaginal discharge. In the chronic variety, the granules are "either small, hard, and whitish—reddish and soft—or miliary, without redness of the surface of the cervix uteri from which they grow"—and without the excessive vascularity of the acute form.

The treatment consists in local bloodletting, warm baths, injections, and counter-irritation, in the acute stage: in the chronic form, astringent injections and counter-irritation externally; tonics and the mineral waters internally.

### VI. Inflammation of the Mucous Membrane of the Uterus; or, Uterine Leucorrhœa.

We have already noticed the corresponding affection of the vagina; the distinction insisted upon by Dr. Churchill between vaginal and uterine leucorrhœa, leading as it does to very important practical differences in the treatment of the two diseases, has nevertheless been little noticed by previous writers on female complaints. Siebold and Joërg, among the Germans, describe the uterine variety; and Boivin and Dugès, among the French, allot a chapter to it. Dr. Locock withdraws from the attempt to establish such a distinction in despair; Dr. Blundell treats of vaginal leucorrhœa only; while the French writers generally restrict the term leucorrhœa to the uterine discharge. Denman, Hamilton, Burns, and Marshall Hall, among our own countrymen, very accurately distinguish the two seats of the respective complaints. An examination with the speculum, by M. Marc d'Espine, *(Archiv. Gen. de Med. Feb.* 1836,) gave the following result in 193 cases. In 130, the discharge was abundant—in 40, a drop only of discharge at the orifice—in 23, the orifice was dry. The orifice may be healthy, pale, red, bright red, or granulated and bloody.

Dr. Churchill enumerates the following circumstances under which it occurs, as "not only illustrative of its nature," but also "as affording *data* for our diagnosis."

1. In young delicate females, "at one or two of the monthly periods preceding the development of the catamenia, and vicarious of them."

2. In suppressed menstruation, "the monthly periods are often marked by a discharge of 'whites,' nearly the same in quality, and continuing as long as the natural secretion."

3. The *intervals* of menstruation being occupied by uterine leucorrhœa, the discharge increasing for two or three days before the appearance of the menses, and when these have subsided, returning in great quantity. It not unfrequently supersedes and becomes vicarious of the catamenia.

4. Menorrhagia is occasionally caused, and sometimes accompanied by it; the complication adding much to the distress of the patient, and the menorrhagia not being easily relieved until the leucorrhœa has been cured.

5. The few last periods, previous to the cessation of the menses, are often characterized by this discharge occurring as vicarious of, or alternately with, the proper menstrual flux.

6. In chlorosis it is often vicarious of the menses, and continues so for many months.

7. After abortion, it is "secreted either constantly or occasionally, for some months, and this condition of the uterus appears to predispose to successive abortion."

8. After the disappearance of the lochia, consequent upon child-bearing, this discharge will often continue for several weeks; or, at the end of one and two months after a first confinement, it frequently appears to the great but unnecessary alarm of the patient and her friends.

"These," says our author, "are the principal circumstances under which I have observed the disease, and in which little doubt can be entertained as to the source of the discharge. *In all the varieties it exists either concomitantly with, or immediately succeeding to, an evident uterine affection, or it is complicated with menstruation.* In the former, there is an *à priori* presumption, that the discharge is from the uterus, and in the latter, the effects of the periodical determination of blood to that organ, upon the quantity of the secretion, would seem to point to a similar inference, especially when we find that *no such augmentation is observed in vaginal leucorrhœa.*

At the same time, it cannot be denied that vaginal leucorrhœa may be also present in any of the foregoing cases, although the uterine disorder be predominant, and modify all the symptoms. Neither is it asserted, that all cases are as obvious, and as easily to be made out, as it would appear from the description on paper.

It may be defined as *a more or less profuse discharge of fluid secreted by the lining membrane of the uterus, (in a state of inflammation,) varying a good deal in quantity and colour, but neither accompanied nor followed, necessarily, by disorganization of the tissue of the womb.*

It may attack females of all ages, the *acute* form is more frequent in younger, the *chronic* in elder persons. It is observed in women of every temperament, according to the peculiar cause. In the leuco-phlegmatic, in whom, from deficient '*materiel,*' the uterus appears unequal to the secretion of the florid catamenia, or in whom, from constitutional causes, the vessels of the mucous membrane lining the womb are in a state of unusual activity: in the plethoric and robust, in whom the circulation, rapid and energetic throughout the whole system, is peculiarly so in the sexual organs during their functional life;—and in the melancholic, whose mental depression so frequently aids in the aggravation of what was originally a trifling malady, and whose fears are acutely alive to any disorder affecting these parts."—*Churchill,* 129-30-31.

The causes of this disease are the same as those enumerated under the previous heads. Dr. C. thinks it may arise also, in part, from certain states of the constitution.

It may be either *acute* or *chronic*. Of a very severe type of the former, the following is a description by M. Lisfranc, (*Mal. de l'Uterus*, p. 249.)

"Often, after some inappreciable cause, an unpleasant itching of the genitals is felt, increasing until it reaches to the uterus, to this is joined a sense of heat and weight in the pelvis. The hypogastrium becomes tense and sensible to the touch. The womb seems to press inconveniently upon the perineum. The patient experiences dragging about the loins, extending to the groins, hips, sacrum and thighs. There is frequent desire to pass water. The pudendum often participates in the tumefaction of deeper-seated parts, and hence standing and moving is very painful; and if the swelling of these parts is considerable it may be impossible to remain in a sitting posture. This state is ordinarily accompanied by nausea, lassitude, and 'malaise,' sometimes by pain in the joints. About the third or fourth day, if the disease be not previously arrested by appropriate treatment, a clear, limpid, viscous discharge escapes from the vulva."

When the menstrual has been superseded by the leucorrhœal discharge, the effects are necessarily severe. The local suffering is great, being indicated by constant itching or pain in the uterus. The constitution of the sufferer sympathises; she is languid, and indisposed to move or exert herself; the pulse *acquires* and accelerates its speed, while its volume diminishes; it is, *as in all cases of inflammatory uterine affections*, hard and incomprehensible; her skin assumes the chlorotic colour, flabbiness, and clammy moisture, although it is sometimes hot and dry; her eyes sink in their orbits, and their lids become discoloured and livid. Frequent and severe nervous headaches, in the region of the occiput principally; vertigo and faintings; sympathetic pains in the kidneys, spleen, and other remote parts; and palpitations of the heart;—accompany the local disease, and form very characteristic marks of its true character.

"The tongue is seldom dry or loaded, but generally of a yellowish red colour, flabby and indented by the teeth." (Marshall Hall). And a diminished and fastidious appetite, torpid bowels, deficience of bile, and the forehead and face spotted with acné punctata vel rosacea, sum up the melancholy catalogue of symptoms.

The discharge varies in quantity, and in quality. Dr. Churchill has known it "so profuse as to oblige the patient to use several napkins in the course of the day." In most cases it is bland, unirritating, and colourless; in some, however, it has been observed by Dr. Hamilton, sen., of a greenish or brownish tinge; by Dr. Churchill, so acrid, as to excoriate the labia and adjacent skin.

It is of variable duration. But, happily, almost universally curable. As it may be confounded with, so must it be distinguished from, 1st, uterine gonorrhœa; 2nd, vaginal leucorrhœa; 3rd, inflammation of the glandular apparatus of the cervix uteri; 4th, from the contents of an abscess of the uterus, ovary, or cellular membrane, discharged through the vagina. The diagnosis, though difficult, may be obtained by attention to the following particulars. In gonorrhœa there are generally present the superficial erosions described by Ricord—a burning pain along the genital canal—a deeper-coloured discharge than in leucorrhœa—scalding on voiding the urine—and urethral discharge—"when uterine leucorrhœa occurs during the intervals of menstruation, the discharge is always increased after the catamenia cease, and most frequently before they appear, and gradually encroaches upon the due performance of that function, rendering the flow less copious or less regular." *Churchill*, p. 136. As far as his experience goes, Dr. C. adds, "no such phenomena occur with vaginal leucorrhœa." In inflammation of the glandular apparatus of the cervix uteri there is a regular, white opake discharge, and constant tenderness on pressure. In abscesses, there can be no mistake, if ever so little attention be paid to the qualities of the matter discharged, and to the presence or absence of previous symptoms of uterine leucorrhœa.

The difference in the treatment of vaginal or uterine leucorrhœa, rests chiefly in the use of injections, which, decidedly beneficial in cases of the former disease, are as decidedly injurious in cases of the latter. The catalogue of remedies reported to have been prescribed with success in this last disease, is a long one. Balsam of copaiba; sulphate of iron; log-wood; spurred rye; powdered colchicum root; iodine; capsicum; quinine; the chalibeate waters; tepid or cold salt water effusions; blue pill and rhubarb; aloes; assafœtida and castor oil; emollient enemata; conium; hyosciamus and opium; water or milk and water ablutions; and lotions of sugar of lead and water, and of calomel and lime water; like the pots, bottles, and boxes in the needy apothecary's shop, who sold the Mantuan drug to Romeo— "all thinly scattered, to make up a show," have been severally recommended in this disease. We object very greatly to such thronged lists of remedies for each particular complaint. They display, not so much the resources of the physician, as the indefiniteness of his knowledge of the specific properties of medicines. Dr. Churchill has seen benefit derived from the four first mentioned, and has found their beneficial effect greatly increased by the previous application of a blister to the sacrum.

### VII. Inflammation of the Substance of the Unimpregnated Uterus.

This is both a rare and an obscure disease, occupying the body, or cervix, or both; and either confined to the proper tissue of the uterus or involving its lining membrane: and seldom occurring before puberty or even before marriage.

If acute, there is pyrexia at the commencement, succeeded by heat and uneasiness in the pelvic region—occasional sharp pains in the back, darting through to the symphysis pubis, and down to the groins and thighs. There is, besides, a constant, dull, bearing-down pain, increased by coughing or sneezing. Pressure of the abdomen downwards, towards the brim of the pelvis, occasions considerable pain. On examination, internally, the womb is found to be enlarged, and pressing the cervix gives pain. In some cases menstruation goes on, but the sufferings of the patient are greatly aggravated at every monthly period. In other cases it is entirely arrested; and, now and then, there is a slight mucous discharge. The constitution is very variously affected. The pulse is quick and small. The skin generally hot and dry, but sometimes also cold and clammy. The local irritation extends to the rectum, vagina, bladder and urethra, and there are pain and difficulty felt in voiding either the fæces or urine. Other parts sympathise with the uterine disorder—the breasts swell and become painful—the stomach is rendered irritable—anorexia and dyspepsia follow in the train—the bowels are constipated, and the general health is impaired.

If *chronic*, the above symptoms are all present; but in a minor degree, and there is but little constitutional suffering, a soft pulse and only slightly accelerated.

It frequently terminates in resolution. Never degenerates into cancer. Is only very seldom fatal. It may, however, terminate in hypertrophy, ramollissement, abscess, and gangrene.

It may be confounded with scirrhus of the womb, cancer, inflammation of the bladder and rectum, and with gastric irritation.

The treatment depends upon the character of the attack. In both, it must be decidedly antiphlogistic. General bloodletting, however, is less to be resorted to than local blood-letting. Guibourt and Duparcque apply leeches to the uterus itself by means of the speculum, and the practice is deserving of further trial. Dujarrie Lasserve recommends the uterus to be repeatedly punctured—a practice, as utterly to be repudiated. Drs. Marshall Hall and Heming advise counter-irritation. Astruc and Mr. Stewart have used cooling and anodyne enemata with benefit—and, by the latter, they are preferred to the vaginal injections. While, according to Dr. Churchill, anodyne fomentations externally employed, and, at a more advanced stage, embrocations to the loins, are "highly beneficial."

Calomel, antimony, and opium, are invaluable internal remedies.

### VIII. Inflammation of the Fallopian Tubes.

The Fallopian tubes are subject to the same morbid changes as the uterus and ovaries; and, besides this, they participate in all the acute diseases of each. The diagnosis is, from this circumstance, extremely difficult. Happily, however, the treatment does not depend upon their being accurately distinguished from the similar affections of those organs.

Acute inflammation of these tubes, is generally only an extension of the inflammation previously subsisting in the uterus or the peritoneum. It sometimes arises as an idiopathic affection.

Its symptoms are "deep-seated, throbbing pain in the hypogastrium or iliac region, extending to the groins and down the thighs," local heat, abdominal tenderness, and the absence of swelling. Dr. Robert Lee, in the articles "Ovaria" and "Pathology of the Uterus," printed in the Cylopædia of Practical Medicine, has expressed his belief that, "in many cases of painful menstruation there exists a state of great congestion or inflammation of these organs (ovaries), and there can be little doubt that the Fallopian tubes often participate in the same disease." And Boivin and Dugès, attribute to chronic inflammation of the internal, or mucous membrane, alone, the discharge, in many cases of supposed leucorrhœa, whether uterine or vaginal. Melanotic and tuberculous diseases, are, by the latter writers, also traced to the same cause.

Both acute and chronic inflammation may terminate in suppuration, and the abscess open either internally or externally.

Of the existence of chronic inflammation of the Fallopian tubes, there can be no doubt. The diagnosis, in the present state of our science, is, however, impracticable.

Adhesions of their fimbriated extremities to the ovaries, and obliteration of their canals, are frequent consequences of such inflammation—and of course occasion sterility in the sufferers. " One of the most frequent morbid appearances," says Dr. R. Lee, " observed in the bodies of young subjects after death, is adhesion of the Fallopian tubes to the ovaria, by short, firm, adventitious membranes; or by long, slender, transparent filaments." —*Cyclop. of Practical Medicine*, vol. ii. p. 377.

The indications of treatment are the same as in metritis.

### IX. Inflammation of the Ovaries.

Inflammation of one or both ovaria is sometimes, though but rarely, an idiopathic disease. More generally, however, it is complicated with peritoneal or uterine inflammation. The entire substance of the ovary is, in most cases, involved in the morbid action—although, by some, it is supposed to affect, in particular cases, only the Graafian vesicles.

The periods of its most frequent occurrence are, immediately prior to, at, and shortly after menstruation; and, shortly after abortion or labour.

The symptoms are always more or less fever; a hot skin; and accelerated pulse; deep-seated and severe pain in the cavity of the pelvis; accompanied with a sensation of burning; the pain not constant, if the patient remain quiet—aggravated if she move about. A slight puffiness or swelling may be perceived on either or both sides of the abdomen at its lower part, which is painful when pressed. An examination " per vaginam" elicits nothing, while, on the other hand, one "per rectum" enables the examiner to reach the ovary, and to ascertain whether its bulk be increased, and whether it be tender under pressure. Dr. Löwenhardt first pointed out to the profession the importance and accuracy of the information obtained by this latter examination.

The acute sometimes issues in the chronic form. Both may terminate in resolution. Or the inflammation may spread to the broad ligaments and to the peritoneum generally. Softening of the substance of the ovary may also result from this disease—or it may run into suppuration, and in these last cases enormous quantities of pus have been deposited; the formation of which is indicated by rigors, softness of the pulse, mitigation of constitutional symptoms, and increasing sense of local weight and throbbing. Gangrene, of course, is a fatal termination, but it seldom takes place. Swelling and induration may likewise be terminations of the chronic form. "Such cases, after the commencement of the disease, will often remain stationary, and without any inconvenience for many years."—*Dr. Seymour, Diseases of the Ovaries*, p. 40.

In both forms of the disease, the treatment is the same in kind, and differs only in degree, being proportioned to the intensity of the symptoms.

" In the acute form, * * * * The most active antiphlogistic treatment will be necessary, venesection, leeches to the iliac region, to the groins, anus, or labia, should be prescribed, (*Solon*), followed by poultices and fomentations to the lower belly, calomel and opium, &c. Emollient vaginal injections and enemata will be beneficial; absolute rest and a spare diet must be adopted. A judicious application of these remedies will, in many cases, especially in idiopathic ovaritis, be adequate to the relief of the disease.

We must attentively watch the course of the disease, and be prepared to meet each *complication* appropriately.

If matter be detected in the iliac fossæ or groins, it must be evacuated, but it is desirable that we should wait until adhesions be formed between the ovary and peritoneum (*Solon*); whenever this is the case, an opening is to be made (*Boivin* and *Dugès*) with a bistoury or caustic. M. Solon thinks the latter preferable, because it tends to determine adhesions, whilst it forms an eschar, which eschar may be punctured in its centre.

If the pouch of matter be felt through the parietes of the vagina, it will not be difficult to penetrate it with a lancet or trocar. In a case, related by M. Solon, which occurred in the hospital Beaujon, absorption of the matter took place just as it was determined to puncture the cyst.

Against gangrene we may employ antiseptics and chlorides internally, with blisters and camphorated frictions externally.

In the *chronic form,* antiphlogistics are no longer of the same value, and we must have recourse to counter-irritation by setons, moxas, &c.

Benefit is sometimes derived from frictions with iodine, or from its combinations with mercury.

Small and repeated doses of calomel have been found very useful, with decoction of sarsaparilla.

The general health should be attended to; the diet must be moderate, and gentle exercise may be taken."—*Churchill,* p. 358-9.

There are two diseases very closely allied to inflammatory affections, which, however, in the one case, by Dr. Gooch, and in both by Dr. Churchill, are looked upon as possessing rather a neuralgic than an inflammatory character. The one is, itching of the vulva—the other, irritability of the uterus. Both are melancholy diseases; the former, because, if neglected, leading to moral depravity; the latter being slow of cure, rendering the sufferer unfit for the duties of a wife, and, unless removed, precluding the possibility of her becoming a mother. We incline to consider them as subacute inflammations—and the success of mild antiphlogistic treatment, in the hands of both writers, confirms us in this view.

The terminations of inflammation are too well known to require enumeration—they are common to every tissue and organ in the body, which is capable of becoming the seat of inflammation, with hardly any exception—and the uterine system is not one of the exceptions. Ulceration is one of these terminations, and we have now to consider this instance of a new disease succeeding to and consequent upon a previous one; in the cases of simple ulceration of the neck of the womb, and corroding ulcer of the uterus; the chancrous ulcers originating in impure connexion, have nothing in them to claim present notice—and the superficial ulceration occurring in gonorrhœa has been already adverted to.

### X. Simple Ulceration of the Cervix Uteri.

This is a disease apparently unaffected by temperament; and occurs at any time after the development of uterine action, but is much more frequent in women who indulge in sexual intercourse than in virgins—and in prostitutes than in married women.

Arising from violence, cold, the use of astringent injections, and the practice of masturbation, its symptoms succeed one another in the following order. Occasional shiverings—flushes of heat in different parts, especially the face—a dragging pain in the loins, and sense of weight at the anus, the pain increasing at every monthly period denote the inflammatory stage, before the commencement of ulceration. Sexual intercourse is attended with pain. Then, a slight sanguineous discharge recurs at intervals. The cervix uteri, if felt, is found tender to the touch; more or less swollen and spongy; and heated. The os uteri is preternaturally open.

The ulcers, at the beginning, are very numerous and small, but afterwards coalesce, and vary in size "from a pin's head to a shilling." Their surface is reddish, and their edges well defined. They are of varying depth, in some instances being mere erosions—in others, involving the whole substance of the cervix.

They may be distinguished from the syphilitic by their regular and defined edges, and by the difference of the discharges in the two diseases. In lues, it is yellow; in the simple ulcer, dirty white, occasionally bloody, and inodorous.

The corroding ulcer and the cancerous ulceration are as easily distinguishable from it: in the former of these two all the symptoms are more severe; there is concomitant hæmorrhage, and a stinking discharge. In the latter, morbid deposition, immobility of the uterus, a fetid discharge, and the peculiar gnawing pain of cancer.

In the inflammatory stage the disease may be subdued by antiphlogistic treatment. In the ulcerative stage, warm emollient injections and astringent ointments are both recommended: in the event of their failure, the repeated application of caustic to the ulcerated surface. Jobert and Marjolin are reputed to have had great success in the management of these cases; the remedy they apply is the pernitrate of mercury.

The bowels are of course to be kept empty. And the frequent use of the hip-bath will always prove beneficial. And here, it occurs to us to recommend in all cases of inflammation and irritation of the uterus the evacuation of the bowels, not by purgatives received into the mouth, but by emollient glysters. In the former, the effect is produced by irritation—that irritation is propagated by sympathy to the uterus, and the disease already there is increased. By the latter, the hardened fæces are simply removed, the gut merely emptied, not irritated. And irritation is taken away instead of being added.

Dr. Churchill asks—"Should these remedies fail, are we under any circumstances to excise the neck of the uterus?"—Certainly not. We have had on this side of the Channel, French surgery crammed into us, usque ad nauseam: and are happy to say, the operation for excising the neck of the uterus is a piece of French surgery. Lisfranc's book makes it appear "a simple and safe operation"—*simple* enough in all conscience it certainly is—how far it may be considered *safe* even in the hands of the *veritable* Lisfranc himself, is to be learnt, not from that chirurgeon's book of fables, but from the complete exposure of his mis-statements by M. Pauly.

"1. Instead of the 99 operations stated by M. Lisfranc to have been performed by him, only 53 can be made out.

"2. There are no exact accounts of the failures which happened in hospital.

"3. Out of 19 private patients operated upon, only one has been permanently benefitted.

"4. Of these 19 cases, four died within 24 hours—twelve had an immediate relapse, and in two others, the carcinoma not being entirely removed, the patient only sank the more rapidly.

"5. Out of nine patients operated upon under M. Pauly's observation, and near whom he remained 24 hours, six were attacked with frightful hæmorrhages; and of these six, three died within 24 hours."

Well may Dr. Churchill add:—"*Such facts are enough to deter the most hardy from attempting this fearful operation, and* THE EXPOSURE OF SUCH MIS-STATEMENTS IS A STRIKING LESSON TO ALL WHO, IN ORDER TO MAKE A REPUTATION, ARE READY TO FORSAKE THE PATHS OF HONOUR AND TRUTH."—P. 248.

We presume that, after the above exhibition of veracity, we shall find few English surgeons so infected with the cacoethes operandi as to perpetrate M. Lisfranc's operation—"*so* simple and *so* safe."

We pass on to another and more terrible disease.

### XI. The corroding Ulcer of the Uterus.

A disease frequently confounded with, but essentially different from, cancer; and liable to be neglected by incautious observers, if mistaken for simple ulceration of the uterus.

It attacks females of the "lymphatic temperament" especially; and occurs when the menses cease, or shortly after.

Its symptoms are frequently ushered in by occasional pain or uneasiness in the pelvis—an internal sense of heat—and leucorrhœa. Sometimes there are no precursory signs, and the first indication of disease is a profuse hæmorrhage, when, upon examination, more or less ulceration of the cervix uteri is found, having a rough granular surface, and differing in sensibility in different cases. "*The remaining portion of the uterus is scarcely at all enlarged, and the contents of the pelvis are free and moveable.*"—*Churchill*, p. 212.

The hæmorrhage returns at intervals, diminishing in quantity towards the close of the disease, and affording temporary relief. In the meanwhile, a profuse discharge from the vagina takes place, of a thin, ichorous, and fetid matter, varying in colour from a pale yellow to a dark brown. The patient complains of weakness, weight, and pains in the back, loins, and lower belly—sometimes lancinating, at others burning. The constitution becomes seriously implicated; and diminished appetite, occasional sickness, irregular action of the bowels, a hurried but feeble circulation, a dry skin and sallow complexion, with low fever, render miserable the existence of the sufferer.

The progress of the disease may be slow, or it may be rapid. During it, occasional examinations *per vaginam* discover the ulceration extending itself circularly round the cervix uteri, or on the surface of the uterus, and, at length, penetrating the bladder, or rectum, or both. Excoriation of the vulva often aggravates the disease. And the patient at last sinks from exhaustion, or may be carried off by peritonitis or by hæmorrhage.

After death, "the uterus is found more or less destroyed by ulceration, which sometimes extends itself circularly, so as to destroy the cervix and part of the body completely, leaving the remainder suspended by the ligaments, and unconnected with the vagina, except by the surrounding cellular tissue; in other cases, it attacks the anterior or posterior wall of the uterus only, with the neighbouring portion of the vagina, and the bladder or rectum. If the bladder be perforated, the vagina will be found more or less coated with matter deposited from the urine: if the communication be with the rectum, fæcal matter will be found in the vagina: I have never seen a case in which the bladder and rectum were both perforated. It is important to remark, that there is no deposition of new morbid matter either in the uterus itself, or in the neighbouring parts."—*Churchill,* p. 213-14.

Sir Charles Clarke says there will appear abundant evidences of the destructive process, but no hardness, no thickening, no deposit of new matter.

The similarity of this disease to cancer is very great—the diagnosis proportionately difficult—a vaginal examination alone can detect the distinguishing mark. *In cancer* there is considerable "deposition of new matter into the cellular membrane and glands between the vagina and rectum—and between the vagina and bladder." *In corroding ulcer,* no such deposition takes place. *In cancer,* the uterus is immovable. *In corroding ulcer,* it may be moved by the gentlest touch.

The prognosis is generally unfavorable—and little therefore can be hoped for from remedies, but a palliative effect. Excision of the cervix uteri is a barbarous, unsafe, and most uncertain operation. Extensive cauterization might possibly be useful. Dr. Churchill has used vaginal injections of nitrate of silver, of the strength of 10, 20, and 30 grains of the salt dissolved in two or three ounces of water, with temporary benefit; the pain being assuaged, and the discharge rendered inodorous.

And here, for the present, we finish our notice of Dr. Churchill's valuable compendium; but only for the present—hoping to bring it a second time before our readers, when we review the subject of female diseases; and in the meanwhile strongly recommending it to every practitioner, as a volume which will amply repay its purchasers and readers.

It is one of the best medical digests with which we are acquainted, and its author evidently combines the erudition and good taste of the scholar and the gentleman, with the professional experience of an accomplished physician. His language is all that can be desired in professional writing—and the candour with which he notices contemporaneous writers who have gone over the same ground with himself, reflects credit alike upon his head and heart. And it is well—for if any man can afford to praise the labours of others it is surely he whose own labour is deserving of all praise. The professional public, which is generally a discriminating one, will, we trust, confirm our verdict, by speedily calling for a second edition of Dr. Churchill's work. We look forward with much pleasure to the announcement of another volume on the Diseases of Females during Gestation, from the same pen; and hope the success of this, will accelerate the publication of that.

